# Nrf2 Overexpression Decreases Vincristine Chemotherapy Sensitivity Through the PI3K-AKT Pathway in Adult B-Cell Acute Lymphoblastic Leukemia

**DOI:** 10.3389/fonc.2022.876556

**Published:** 2022-05-12

**Authors:** Li Wang, Xin Liu, Qian Kang, Chengyun Pan, Tianzhuo Zhang, Cheng Feng, Lu Chen, Sixi Wei, Jishi Wang

**Affiliations:** ^1^Clinical Medical College, Guizhou Medical University, Guiyang, China; ^2^Department of Hematology, Guizhou Province Institute of Hematology, Guizhou Province Laboratory of Haematopoietic Stem Cell Transplantation Centre, Affiliated Hospital of Guizhou Medical University, Guiyang, China; ^3^Center for Clinical Laboratories, The Affiliated Hospital of Guizhou Medical University, Guiyang, China; ^4^National Clinical Research Center for Hematologic Diseases, The First Affiliated Hospital of Soochow University, Jiangsu, China

**Keywords:** nuclear factor erythroid 2-related factor 2, B-cell acute lymphoblastic leukemia, chemotherapy resistance, Vincristine, BAD, PI3K-AKT pathway

## Abstract

Uncontrolled proliferation is an important cancer cell biomarker, which plays a critical role in carcinogenesis, progression and development of resistance to chemotherapy. An improved understanding of novel genes modulating cancer cell proliferation and mechanism will help develop new therapeutic strategies. The nuclear factor erythroid 2-related factor 2 (Nrf2), a transcription factor, decreases apoptosis when its expression is upregulated. However, the relationship between Nrf2 and Vincristine (VCR) chemotherapy resistance in B-cell acute lymphoblastic leukemia (B-ALL) is not yet established. Our results showed that Nrf2 levels could sufficiently modulate the sensitivity of B-ALL cells to VCRby regulating an apoptotic protein, i.e., the Bcl-2 agonist of cell death (BAD). Chemotherapeutic agents used for the treatment of B-ALL induced Nrf2 overactivation and PI3K-AKT pathway activation in the cells, independent of the resistance to chemotherapy; thus, a potential resistance loop during treatment for B-ALL with a drug combination is established. Therefore, B-ALL patients with a high expression of Nrf2 might mean induction chemotherapy with VCR effective little.

## Introduction

Acute lymphoblastic leukemia (ALL) continues to be one of the most difficult-to-treat cancers in adults, particularly in terms of complete remission rates achieved using conventional therapeutic regimens. Leukemia requires a minimum of 20% blast cells in the bone marrow (BM). The incidence of ALL in adults is significantly lower, representing only 0.2% of all cancers. The prognosis is not encouraging, with an average 5-year survival rate of 20% to 40% (1–3). B-ALL accounts for approximately 75% of ALL cases. Historically, adult B-ALL patients have been associated with a poorer prognosis than pediatric B-ALL patients (4–6). In the past 20 years, treatment algorithms similar to those for children have been developed for adults with B-ALL considering the fundamental principles of induction, early intensification, consolidation, and the presence of complications in the patients (7). Traditional chemotherapeutic approaches that include VCR, corticosteroids, and anthracyclines may not be as effective for most adults with ALL, who may also have multiple comorbidities (8). However, a previous study showed that a few adult patients completed treatment with these traditional chemotherapeutic regimens (9).

Nuclear factor erythroid 2-related factor 2 (Nrf2) is an important transcription factor and a modulator of cellular antioxidant responses, which regulates the expression of genes encoding antioxidant enzymes and exerts a protective role against a variety of oxidative changes (10, 11). Kelch-like ECH-associated protein 1 (Keap1) promotes the ubiquitination and proteasomal degradation of Nrf2, which maintains low basal levels of the Nrf2 protein (12). However, when cells are exposed to oxidative, xenobiotic, or electrophilic stress, Keap1-mediated degradation of Nrf2 is reversed (13). Subsequently, the accumulation of Nrf2 in cells increases (14). Cells have hierarchical responses to defense changes of oxidative stress and Nrf2 activated by a little increase of reactive oxygen species (ROS)/reactive nitrogen species (RNS) at the first time. With this kind of stimulated Nrf2 activated can provide apoptosis response to excessive ROS levels. In other words, Nrf2 activation cause pro-apoptotic signaling (15, 16). Previous studies have shown that the constitutive stabilisation and activation of Nrf2 is associated with poor prognosis in various human cancers, such as head and neck cancer (17), gastric cancer (18), lung cancer (19), esophageal squamous cell carcinoma (20), gallbladder cancer (21), breast cancer (22)and hepatocellular carcinomas (23, 24). Recent studies have reported additional functions of Nrf2, such as effects in drug metabolism and excretion; autophagy; proteasomal degradation; metabolism of energy, iron and amino acids; cell survival and proliferation;

Apoptosis is an active intracellular cell death virtually observed in all higher eukaryotic cell types and is abnormally repressed in many human disorders, such as tumor formation and treatment resistance in tumor cells (25). Phosphatidylinositol 3-kinase (PI3K) is a lipid kinase that controls various cellular activities by spreading intracellular signal cascades (26). In cancer treatment, PI3Ks are thought to be important causes of chemoresistance (27, 28). Protein kinase B (AKT) is another important downstream effector of PI3K signalling, modulating various pathways, including apoptosis suppression, cell growth promotion and cellular metabolism control. Therefore, the PI3K-AKT pathway is important for chemoresistance and is a hub that influences chemoresistance by inhibiting of apoptosis (26, 29). Several studies have reported that PI3K and AKT affect the apoptosis program by regulating apoptosis-related factors (30, 31). In apoptotic pathways induced by several foreign PI3K/AKT stimuli, the balance between Bcl-2 family proteins should be maintained (32). The Bcl-2 family members are antagonised by the varied pro-death proteins (e.g. BAX and BAK) or sequester BH3-only proteins, i.e. BAD (33–37). The cells’ susceptibility to apoptotic stress is regulated by balancing these opposing apoptotic proteins.

Activated AKT inhibits apoptosis by phosphorylating Ser136 of BAD (the AKT target) and releasing of BAD from the Bcl-xL complex (36, 38).

Therefore, this study aimed to determine the role of Nrf2 in chemotherapeutic drug resistance among adult patients with B-ALL. Furthermore, by indirectly regulating the Bcl-2, Nrf2 inhibited the BAD protein expression through the PI3K-AKT signalling pathway, resulting in drug resistance in B-ALL cells.

## Materials and Methods

### Patient Samples and Stable Cell Lines

The research employed B-ALL cells from patients at the Affiliated Hospital of Guizhou Medical University in 2019 and 2020. The samples were separated into three subgroups: “Normal donor” (n = 15), “Drug sensitive” (n = 19) and “Resistant” (n = 14). Patients with B-ALL are classified as “Drug Sensitive” if they obtain CR with standard induction treatment, or “Resistant” if they do not reach CR. Western blot and qRT-PCR were used to evaluate Nrf2 protein and mRNA levels in various subgroups. The patients’ conditions were diagnosed using cytomorphology, cytochemistry, and immunophenotyping. analysed B-ALL samples were taken following hemolysis of red blood cells at diagnosis, pre-treatment, and post-induction. Then, total RNA and protein were isolated from the generated B-ALL cells and utilised for drug testing. Patients with B-ALL were classed as “drug responsive” or “resistant” to treatment. **Table 1** provides detailed patient data. The Ficoll (Solarbio Science & Technology, Beijing, PRC) density centrifugation was used to separate BMNCs from healthy donors and B-ALL patients.

**Table 1:** The patients with B-ALL samples characteristic.

Parameter	N	%
Gender (n = 33)		
Male	18	54.55
Female	15	45.45
Age (year)		
18-34	8	34.78
35-64	23	69.70
≥65	2	6.06
White blood cells count (cells ×10^9^/L)		
<30	16	48.48
30-99	5	15.15
≥100	2	6.06
BCR-ABL (positive)	6	18.18
MLL-AF4	4	12.12
Chromosomal karyotype		
t (9;22)	1	3.03
t (4;11)	1	3.03
Normal	31	93.94
Immunophenotype		
Common-B-ALL	32	96.97
Pre-B-AL	1	3.03

Human B-ALL stable cell lines Nalm-6, Sup-b15, and RS4:11 were obtained from Guizhou Province Laboratory of Haematopoietic Stem Cell Transplantation Center. The cell lines were cultured in RPMI-1640 medium supplemented with 10% fetal bovine serum, penicillin (100 units/ml), and streptomycin (100 mg/ml) at 37°C in a humidified atmosphere with 5% CO2.

### Reagents and Antibodies

Anti-Nrf2 (ab89443) purchased from Abcam (Cambridge, UK). Anti-β-actin (20536-1-AP), anti-BAD (10435-1-AP), p-CREB (12208-1-AP), LMNA (10298-1-AP) antibodies were obtained from Proteintech Group Co., Ltd. (Wuhan, PRC). Anti-AKT1(K101311P), anti-p-AKT1(K006214P), PI3K(K106692P), p-PI3K(K006379P) antibodies were obtained from Solarbio Science & Technology(Beijing, PRC). Anti-BCL-2(AF6139), Cleaved-Caspase 3(AF7022), Caspase 3(AF6311), Cleaved-Caspase 9(AF5240), Caspase 9(AF6348) antibodies were obtained from, Affinity Biosciences (USA). Vincristine (VCR) were purchased from Topscience (Shanghai, PRC). Daunorubicin (DNR) and brusatol (an Nrf2 inhibition) were purchased from MCE (NJ, USA). The chemical inhibitor MK-2206 (MCE, NJ, USA) of the AKT (39). Fetal bovine serum and RPMI 1640 medium were obtained from Gibco (Carlsbad, CA, USA). Nuclear Protein Extraction Kit were obtained from Solarbio Science & Technology (Beijing, PRC).

### Western Blot Analysis

Protein lysate was extracted from cells using RIPA lysis buffer supplemented with 1 μM PMSF (Solarbio Science & Technology, Beijing, PRC) agitated at 4°C for 30 min. The extracts were centrifuged at 12, 000 rpm for 15 min at 4°C, and the supernatant was collected. A BCA protein assay kit (Pierce, Hercules, CA, USA) was used to determine the protein concentrations. Protein (40μg) were then loaded on 10% SDS–PAGE gel, and the separated proteins were transferred onto PVDF membranes. Membranes were routinely blocked in 5% nonfat milk in PBS for 2 h with agitation and washed. Then, the membrane was blotted with primary antibodies for 2 h. After washing, the membranes were incubated with secondary antibodies for 45 min at room temperature. All protein bands were visualised with the use of the enhanced chemiluminescence (7Sea Biotech, Shanghai, PRC). The stable Nalm-6 and RS4:11 cell lines expressing LV-Nrf2 or si-Nrf2 were treated with MK-2206 (10 μM) in RPMI-1640 medium supplemented with 10% FBS for 24 h.

### Quantitative Real Time-Polymerase Chain Reaction (qRT-PCR)

According to the manufacturer’s instructions, total RNAs from cells were extracted using Trizol reagent (Invitrogen, Carlsbad, CA, USA). Real-time PCR was performed using the SYBR Green PCR Master Mix (TianGen Biotech, Beijing, PRC) and the PRISM 7500 real-time PCR detection system (ABI, USA). The following human primers (Generay Bioteach Co. Ltd, Shanghai, PRC) were used in this study:

β-actinF,5´-CTACCTCATGAAGATCCTCACCGA-3´;

β-actin R, 5´-TTCTCCTTAATGTCACGCACGATT-3´;

Nrf2 F, 5´-TGACAATGAGGTTTCGGCTACG-3´;

Nrf2 R, 5´-GGAGAGGATGCTGCTGAAGGAATC-3´;

### Immunofluorescence and Immunohistochemical (IHC) Staining

Immunofluorescence was used to analyse Nrf2 subcellular localisation. Paraformaldehyde (4%), 4 hours, 4 temperatures. After three PBS washes, cells were permeabilised for 15 minutes with Triton X-100. After three PBS washes, cells were blocked using blocking buffer (PBS + 5% BSA) for 60 minutes and then rinsed with PBS (three times). On the third wash, cells were labeled with rabbit anti-human Nrf2 overnight. After that, the cells were either labeled with FITC-conjugated secondary antibody or phalloidin-tetramethyl rhodamine for 45 minutes. DAPI stained nuclei (4-6-diamidino- 2-phenylindole). Fluorescence microscopy was used to image cells. ICC staining required 30 minutes of formaldehyde fixation of B-ALL mononuclear cells. Three times with PBS. The cells were then penetrated into PBS with 0.1 percent Triton-X 100 for 20 minutes. After 5 minutes of repair, the antigen was sealed with 5% BSA for 1 hour at room temperature. They were then treated at 4°C overnight with dilution (1:250 rabbit anti-Nrf2). 3 PBS washes in 10 min. 1 hour incubation with horseradish peroxide second antibody (1:200) followed by 3 washes with PBS. It was incubated for 10 minutes, rinsed for 10 minutes with PBS, then stained for 1 minute with hematoxylin. Gradient ethanol dehydration, xylene transparency for 5 minutes, and lastly a microscope picture. The IHC tests were graded as described (40).

### Lentiviral Transduction

Human Nrf2 overexpression clone lentiviral particle (L-Nrf2) and human Nrf2- silencing RNA (si-Nrf2) were purchased from Genechem Co., Ltd. (Shanghai, PRC). Transfection of Nrf2 was performed using the manufacturer’s instructions. Cells (Nalm-6 and RS4:11), respectively transfected with empty vector (EV), were used as controls. After expansion and maintenance in RPMI-1640 medium supplemented with 10% FBS for 5 days, stable Nalm-6 and RS4:11 cell lines expressing LV-Nrf2 or si-Nrf2 were selected puromycin (1.5μg/ml and 2μg/ml respectively).

### Combined Drug Analysis

Leukemia cell lines cultures were treated with DNRand VCR in the presence or absence of brusatol, added to each drug solution at fixed combination ratios. Cell viability was determined after 24–48h of treatment by CCK-8 assay as described above. To determine the synergistic, additive, or antagonistic effects of drug combinations, we used CompuSyn software (ComboSyn Inc., Paramus, NJ; www.combosyn.com) based on the method of the combination index (CI) described by Chou.25 Synergy, additivity, and antagonism were defined by a CI <1, CI = 1, or CI >1, respectively. Where indicated for some experiments, B-ALL cell lines have been treated with brusatol for 24 h at a final concentration of 1.5 μM.

### Apoptosis Assay

After incubation, cells were washed in cold PBS and resuspended in buffer containing annexin V–APC and PI according to the manufacturer’s instructions. The cell suspensions were analysed by flow cytometry (FCM) (BD Biosciences).

### Xenografted Tumor Model

Nonobese diabetic/severe combined immunodeficiency (NOD/SCID) mice were purchased from SPF Biotechnology Co., Ltd (Beijing, China). Stably transfected Nrf2 cells that were growing in the logarithmic phase were prepared. Cells were resuspended in PBS at a concentration of 5 × 10^6^ cells/100 μL and then subcutaneously injected into the 5-week-old mice. For *in vivo* VCR treatment, twelve mice were dived into four groups. After the xenografts reached 0.5 cm in diameter, two of the groups were treated with VCR (80 mg/kg/day for 6 days) by intraperitoneal injection (41), the others were treated with PBS. Tumor growth was monitored by measurements of the length and width and the tumor volume was calculated using the equation (L × W^2^)/2. After mice were placed on the platform of BLT In-Vivo Imaging System (BLT Photon Tech., Guangzhou, China), fluorescence images were captured according to the manufacturer’s instructions. Animals were euthanised, tumors were excised, weighed and paraffin-embedded. All experiments on mice were approved by the Institutional Animal Care and Use Committee of Guizhou Medical University, China.

### Statistical Analysis

The GraphPad Prism 7 software was used for creating graphs and for statistical analysis. Normally distributed data were expressed as mean ± standard deviation (x ± s), and a t-test was performed for the intergroup comparison; Paired sample t-test was used to compare the values of the same individual at different time points. A rank-sum test was used to compare the data that did not follow a normal distribution. P < 0.05 was considered statistically significant.

## Results

### Nrf2 Is Overexpressed in Chemotherapy-Resistant Adult Patients With B-ALL

**Figure 1:**
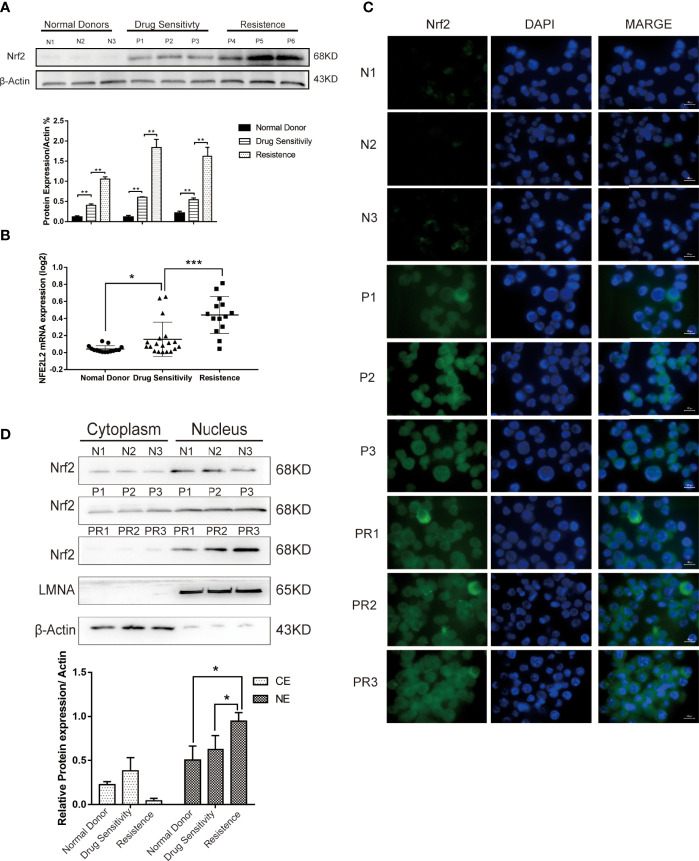
**(A)** Expression levels of Nrf2 protein were detected in 3 donors and 6 B-ALL samples by western blot. **(B)** Quantification of Nrf2 expression in donors (n = 15) and B-ALL samples (Drug sensitivity, n = 19; Resistance, n = 14). Values are presented as mean ± SD (n = 3), *P < 0.05, **P < 0.01, ***P < 0.001. **(C)** Immunofluorescence staining analysis of the expression of Nrf2 in ALL patients (N, normal donors; P, patients of drug sensitivity; PR, patients of resistance). Green indicates Nrf2, and blue indicates DAPI. Scale bars = 10 μm. **(D)** Cytoplasmic and nuclear fractionation kit was used to obtain the cytosolic and nuclear protein. LMNA and β-Actin were considered as nuclear and cytosolic loading control, respectively (N: normal donors, P: patients of drug sensitivity, PR: patients of resistance). And bands were quantified. NE, nuclear extract. CE, Cytosolic extract. Values are presented as mean ± SD (n = 3). Significant differences are indicated by *p < 0.05 vs. Ctrl-NE group.

To determine the potential role of Nrf2 on chemoresistance in B-ALL, we measured the Nrf2 protein and mRNA expression in leukemia cells isolated from the BM of healthy donors and patients with B-ALL (**Table 1**). The Nrf2 protein (**Figure 1A**; P < 0.01) and mRNA (**Figure 1B**; P < 0.001) expression levels were significantly higher in the resistant subgroup than in the drug sensitive subgroup. Moreover, the Nrf2 protein and mRNA expression levels were the lowest in the cells obtained from healthy donors (**Figure 1B**; P < 0.05).

To further investigate the subcellular distribution of Nrf2 expression in patients’ primary B-ALL cells, we used immunofluorescence staining (IF). Our results revealed that the resistant subgroup showed a higher accumulation of Nrf2 in the nuclear and cytoplasm (**Figure 1C**). Furthermore, the nuclear and cytoplasmic accumulation of Nrf2 was higher in cells obtained from B-ALL patients than in healthy donors. In patients with B-ALL, Nrf2 expression was found to be significantly related to the curative efficacy of conventional chemotherapy. The expression of the Nrf2 protein in the nucleus of resistance patients (n = 3) was higher than that in the nucleus of sensitive patients (n = 3) (**Figure 1D**; P < 0.05). Besides, we confirmed that Nrf2 high expression increased the HO-1 protein expression in RS4:11 cells (**Figures S1A, B**).

### Nrf2 Inhibition Sensitises B-ALL Cells to VCR

**Figure 2:**
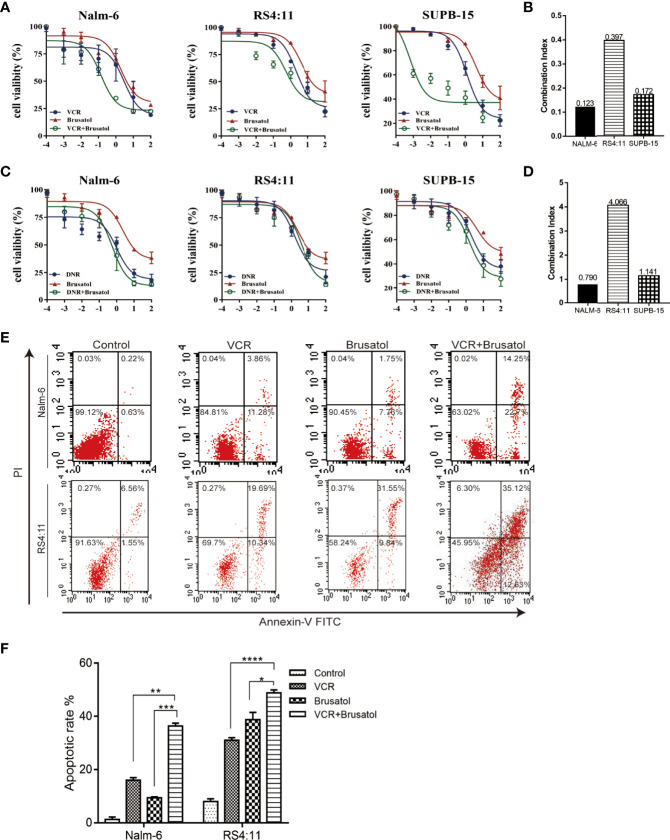
Nrf2 inhibitor, brusatol, sensitises B-ALL cell lines to VCR. **(A)** Dose-response curves of VCR(10^-4^,10^-3^, 10^-2^, 10^-1^, 10 and 10^2^ μM), brusatol (10^-5^, 10^-4^,10^-3^, 10^-2^, 10^-1^ and 10 μM) and VCR (10^-4^,10^-3^, 10^-2^, 10^-1^, 10 and 10^2^ μM) combination at the constant molar ratio with brusatol (1.5μM) in B-ALL cell lines. CCK-8 assay after 24 h of drug exposure determined cell viability. **(B)** Combination index (CI) values were calculated for each drug combination at effective dose ED50. **(C)** Dose-response curves of DNR (10^-4^,10^-3^, 10^-2^, 10^-1^, 10 and 10^2^ μM), brusatol (10^-5^,10^-4^,10^-3^, 10^-2^, 10^-1^ and 10 μM) and DNR (10^-4^,10^-3^, 10^-2^, 10^-1^, 10 and 10^2^ μM) combination at the constant molar ratio with brusatol (1.5μM) in B-ALL cell lines. CCK-8 assay after 24 h of drug exposure determined cell viability. Data are expressed as mean ± S.E.M. of at least three independents’ experiments. **(D)** Combination index (CI) values were calculated for each drug combination at effective dose ED50. **(E, F)** The combined VCR (5 μM)/brusatol (1.5 μM) treatment increases apoptosis in B-ALL cell lines for 24h. An Analysis of apoptosis (by Annexin-V/PI staining) induced by brusatol, VCR, and their combination (at same molar ratios as in A and C) at the indicated concentrations 24 h post-treatment. *P < 0.05, **P < 0.01, ***P < 0.001,****P < 0.0001.

To examine the role of Nrf2 in the phenomenon of drug resistance, we evaluated whether brusatol (an Nrf2 inhibitor) could enhance drug response in connection with cell viability. A western blot showing Nrf2 protein different levels of three B-ALL cell lines (Nalm-6, RS4:11, and Supb-15) and the level expression of Nrf2 in Nalm-6 and RS4:11 is similar. Supb-15 cell line has the highest expression of Nrf2 (**Figures S2A, B**). We used them treated with VCR (10^-4^,10^-3^, 10^-2^, 10^-1^, 10 and 10^2^ μM) and daunorubicin (DNR, 10^-4^,10^-3^, 10^-2^, 10^-1^, 10 and 10^2^ μM), which are drugs used in the standard treatment of B-ALL, and subsequently with brusatol (10^-5^,10^-4^,10^-3^, 10^-2^, 10^-1^ and 10 μM). Results from CCK-8 (cell counting kit-8) experiments indicated that brusatol administration altered the sensitivity of all B-ALL cell lines to VCR treatment (**Figure 2A**) and their combination index (CI) values were<1 (**Figure 2B**). Unlike the results observed with VCR, the combination of brusatol with DNR showed no significant change in sensitivity to DNR (**Figure 2C**); however, brusatol showed a slight increase in the sensitivity of Nalm-6 cells to DNR with a CI<1 (**Figure 2D**). In addition, we assessed the effect of drug resistance mediated by the VCR/brusatol combination by analysing the mechanism of cell death induced by treatment with sublethal doses of the individual drugs. The combination of both drugs 5 μM VCR + 1.5 μM brusatol induced a potent proapoptotic response in B-ALL cells, as seen by a significant increase in the number of apoptotic cells (by Annexin-V/PI staining) (**Figures 2E, F**). Therefore, Nrf2 overexpression decreased the sensitivity of B-ALL to VCR.

### B-ALL Cells With High Expression Levels of Nrf2 Had Higher Drug Resistance to VCR

**Figure 3:**
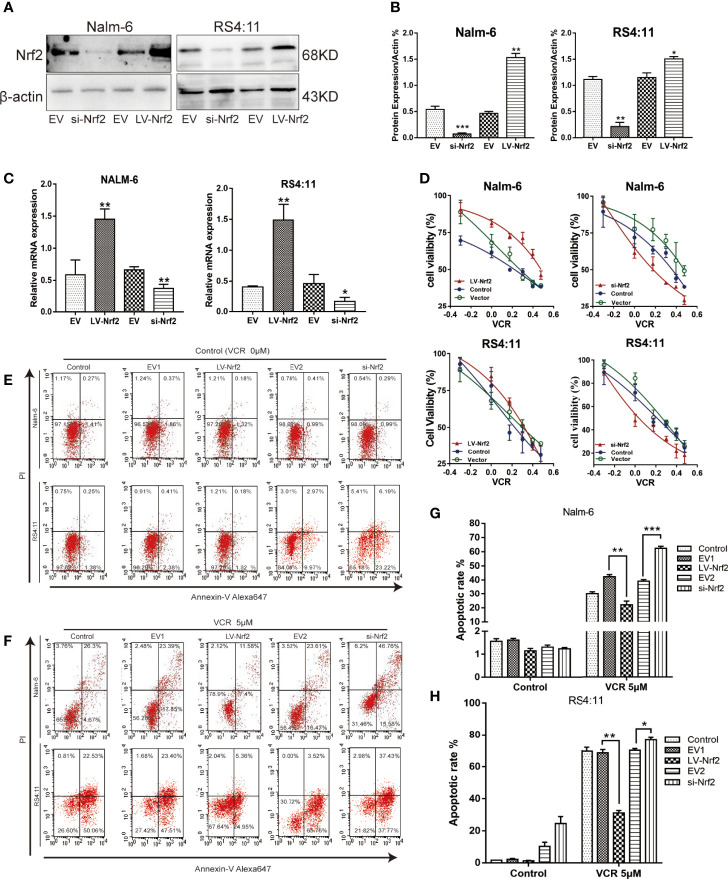
**(A)** Nrf2 was overexpressed or silenced in Nalm-6 and RS4:11 cell lines determined by western blot analyses. **(B)** The relative grey values were shown in the histogram. **(C)** Nrf2 was overexpressed or silenced in Nalm-6 and RS4:11 cell lines determined by qRT-PCR analyses. **(D)** Dose-response curves of VCR (0, 0.05, 0.1, 0.25, 0.5, 1 and 2 μM) of LV-Nrf2 and si-Nrf2 in Nalm-6, RS4:11. CCK-8 assay after 24 h of drug exposure determined Cell viability. Data are expressed as mean ± SEM of at least three independent experiments. **(E, F)** The percentage of apoptotic cells was demonstrated by flow cytometry in both cell lines following the LV-Nrf2 and si-Nrf2 after 24h. **(G, H)** The percentage of apoptotic cells was demonstrated by flow cytometry in both cell lines following the LV-Nrf2 and si-Nrf2 after being treated with VCR (5 μM) for 24h. *P < 0.05, **P < 0.01, ***P < 0.001.

Increased Nrf2 expression has been associated with resistance to chemotherapy in B-ALL. The expression level of Nrf2 in Nalm-6 and RS4:11 cell lines was confirmed using western blot (**Figures 3A, B**) and qRT-PCR (**Figure 3C**). We examined the effect of Nrf2 expression on sensitivity to VCR. Overexpression of Nrf2 *via* lentivirus transduction resulted in reduced sensitivity of Nalm-6 and RS4:11 cells to VCR, whereas the sensitivity to VCR increased upon Nrf2 downregulation (**Figure 3D**). These data suggest that expression of Nrf2 may mediate resistance to VCR in B-ALL. These findings were confirmed using FCM. B-ALL cells overexpressing Nrf2 had reduced sensitivity to VCR treatment and decreased VCR-induced apoptosis (**Figures 3E–H**; P < 0.01). However, knockdown of Nrf2 using siRNA increased the sensitivity to VCR-induced cytotoxicity (**Figures 3F–H**; P < 0.05). These results indicated that Nrf2 expression was directly correlated with sensitivity to VCR treatment.

### Effects of VCR Treatment on PI3K, AKT, Nrf2 and P-AKT Proteins Expressions in the PI3K-AKT Pathway in ALL Cells

**Figure 4:**
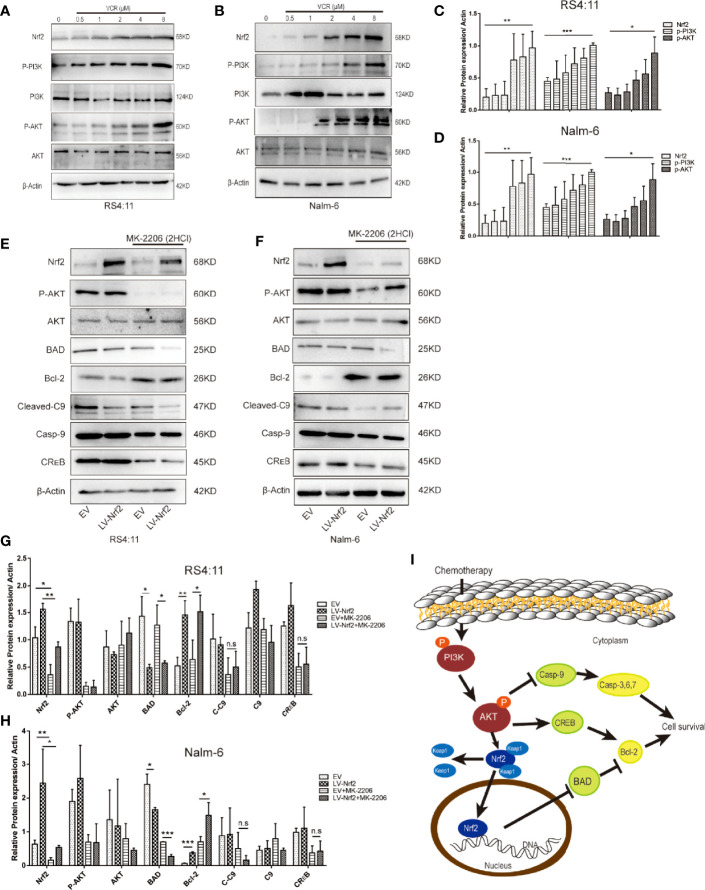
**(A)** Representative western blot of Nrf2, p-PI3K, PI3K, p-AKT, AKT, and β-Actin in RS4:11 cells. The cells were pretreated with or without VCR (0.5,1,2,4 and 8 μM, 24 h). **(B)** Representative western blot of Nrf2, p-PI3K, PI3K, p-AKT, AKT, and β-Actin in Nalm-6 cells. The cells were pretreated with or without VCR (0.5,1,2,4 and 8 μM, 24 h). **(C, D)** The relative grey values were shown in the histogram. **(E)** After treatment with or without 10 μM MK2206 for 24 h in RS4:11 cells, protein expression levels of Nrf2, p-AKT, AKT, BAD, Bcl-2, Caspase-9, Cleaved-Caspase9 and CR_E_B was evaluated by western blot analysis in the Nrf2-overexpression and EV groups. **(F)** After treatment with or without 10 μM MK2206 for 24 h in Nalm-6 cells, protein expression levels of Nrf2, p-AKT, AKT, BAD, Bcl-2, Caspase-9, Cleaved-Caspase9 and CR_E_B was evaluated by western blot analysis in the Nrf2-overexpression and EV groups. **(G, H)** The relative gray values were shown in histogram. Data are presented as the mean ± SD of three independent experiments. *P < 0.05, **P < 0.01, ***P < 0.001, n.s, no significance. **(I)** Schematic representation of the molecular mechanisms proposed in the positive effect of regulate *via* Nrf2 of PI3K/Akt/Nrf2 signaling pathway in ALL cells.

To perform an in-depth investigation of this mechanism, biomarkers of the representative apoptotic signalling pathways, namely, p-PI3K and p-AKT, were detected after treating the cell with or without VCR in RS4:11 cells (**Figure 4A**) and Nalm-6 cells (**Figure 4B**). With VCR treatment, Nrf2, p-PI3K and p-AKT expression levels were significantly increased (**Figures 4C, D**, P < 0.01, P < 0.001 and P < 0.05). This observation confirms that the activation of the PI3K-AKT signal pathway is caused by VCR treatment in RS4:11 cells (**Figure 4E**) and Nalm-6 cells (**Figure 4F**). The role of Nrf2 in drug resistance in ALL cells treated with VCR was further investigated by activating the PI3K-AKT signaling pathway. Western blot was used to detect p-AKT in an empty vector and LV-Nrf2 cell group, and was found that the Nrf2 overexpression did not affect the p-AKT. Next, the protein expression levels of downstream products of this signaling pathway were examined in different groups. The results show that the expression of Bcl-2 in the LV-Nrf2 subgroup was higher than in the EV subgroup (**Figures 4 G, H**; P < 0.01, P < 0.001), and this means that Nrf2 overexpression could downregulate the BAD expression level, increasing the Bcl-2 expression level. Finally, RS4:11 cells (**Figure 4E**) and Nalm-6 cells (**Figure 4F**) were treated with MK-2206 (10 μM) for 24 h, an AKT inhibitor, and LV-Nrf2 showed it decreased BAD levels compared with those in the EV subgroup (**Figures 4G, H**; P < 0.0, P < 0.05). The expression of BAD in the LV-Nrf2+MK-2206 subgroup was lower than it in the EV+MK-2206 subgroup (**Figures 4G, H**; P < 0.05, P < 0.001). The expression of Bcl-2 in the LV-Nrf2+MK-2206 subgroup was still higher than it in the EV+MK-2206 subgroup (**Figures 4G, H**; P < 0.05, P < 0.05). Moreover, p-AKT levels were decreased in both of them, a trend that has not changed.

This study revealed for the first time that increased Nrf2 could reduce ALL cell sensitisation to VCR therapy by repressing BAD in the PI3K-AKT pathway (**Figure 4I**).

### The Nrf2 Expression Is a Directly Associated With the Therapeutic Effects of VCR *In Vivo*

**Figure 5:**
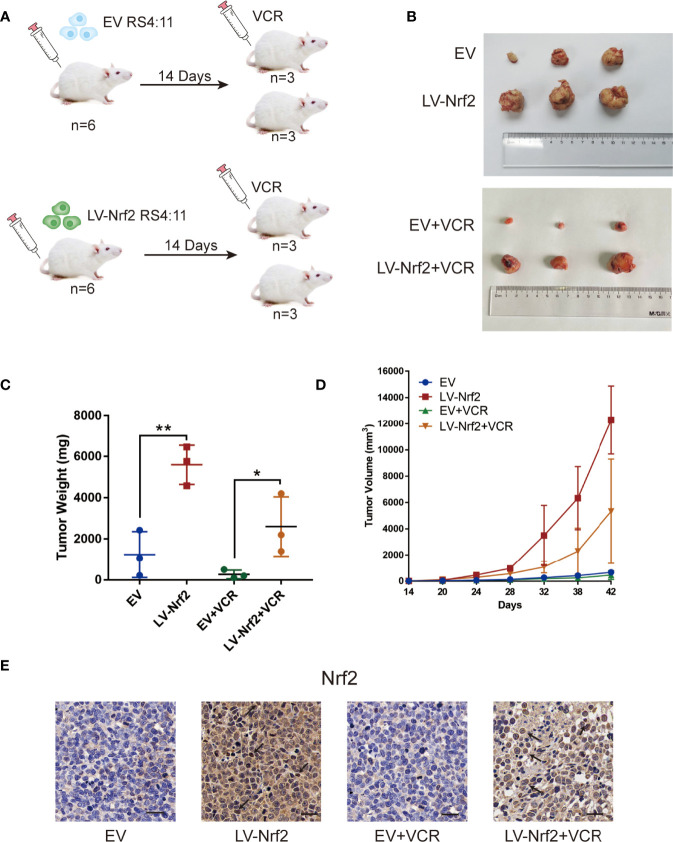
**(A)** Model of mouse subcutaneous tumor formation. **(B)** Images of subcutaneous xenografts from mice in the EV group (n = 3), LV-Nrf2 group (n = 3), EV+VCR group (n = 3) (VCR, 80 mg/kg/day for 6 days), LV-Nrf2+VCR group (n = 3) (VCR, 80 mg/kg/day for 6 days). **(C)** Tumor weight change curves for subcutaneous xenografts. *P < 0.05, **P < 0.01. **(D)** Tumor volume growth curves for subcutaneous xenografts. **(E)** The expression of Nrf2 was examined in xenograft tumor tissue sections using immunohistochemistry (IHC) (scale bars: 50 μm).

A total of 12 B-ALL xenograft models were developed by injecting EV or LV-Nrf2 transfected RS4:11 cells into NOD/SCID mice, as described, to functionally correlate Nrf2 overexpression with the response to tumor cells proliferating, growth and VCR sensitisation (**Figures 5A, B**). Moreover, VCR was administered to the mice as soon as the tumor became palpable. As shown in **Figures 5C, D** tumors in the LV-Nrf2 subgroup (n = 3) were bigger and heavier than those in the EV subgroups (n = 3; P < 0.01). Tumors in the LV-Nrf2/VCR subgroup (n = 3) were bigger and heavier than those in the EV/VCR subgroups (n = 3) (P < 0.05). The tumor volume and weight were significantly reduced in both groups treated with VCR (P < 0.05). Then, an IHC assay was used to look for Nrf2 expression in paraffin-embedded tumor tissues. Neither the Nrf2 overexpression group treated with or without VCR nor the EV group showed any significant differences in Nrf2 *in vivo* (**Figure 5E**). Therefore, NRF2 overexpression is suggested to results in decreased sensitivity of B-ALL cells to VCR chemotherapy.

## Discussion

In recent years, several studies have investigated the potential role of Nrf2 protein, which plays critical roles in carcinogenesis, tumor progression and chemotherapy resistance (35, 42, 43), but not before B-ALL. Therefore, the Nrf2 expression in healthy donors vs. drug-sensitive vs. drug-resistant was analysed in adult patients with B-ALL. The basic case data are fascinating; however, they are mostly hypothesis-generating due to a paucity of longitudinal specimens. Among the main primary patient’s major features, our findings revealed that chemotherapy-resistant B-ALL samples had a considerable Nrf2 overexpression and activation, which was consistent with Nrf2 descriptions in solid tumors (43, 44). Some previous studies demonstrated that Nrf2 transient knockdown or selective inhibition through the Keap1 overexpression strongly increased the susceptibility of lung cancer (44) cells to different chemotherapeutics, including cisplatin (45), doxorubicin (2, 46), and etoposide (24, 47, 48).

In this study, *in vitro* and *in vivo* niche models were employed to further examine the Nrf2 mechanism that induced chemosensitivity and to further understand their functional features in supporting adult B-ALL targets. *In vitro*, we mimicked chemotherapy with medication, employing VCR and DNR alone or combined with brusatol. Our results revealed that brusatol, an Nrf2 inhibitor, sufficiently increased the B-ALL cell sensitivity to VCR through the synergistic therapeutic effects. We recapitulate all these findings in a murine model by activating Nrf2 through tumor resistant VCR therapy. VCR is a primary drug used in induction therapy (49). Previous studies have reported (50) that specific Nrf2 inhibition *via* KEAP1 (23) or Bcl-2 (51) strongly increased the susceptibility of tumor cell lines to different chemotherapeutics, including doxorubicin, cisplatin and etoposide. VCR and anthracycline mitoxantrone are extensively used as standard chemotherapeutics for patients with newly diagnosed and relapsed B-ALL (52, 53) which establishes the high clinical relevance of our results. This sensitisation effect was obtained by specifically inhibiting Nrf2, without the need to counteract additional detoxifying genes, indicating the significant role of Nrf2 family members in modulating drug response in B-ALL. Our findings support the use of brusatol, an Nrf2 inhibitor, combined with VCR as a novel therapeutic approach for the treatment of drug-insensitive or relapsed B-ALL.

Furthermore, gain- and loss-of-function experiments were conducted to investigate the direct impact of Nrf2 in chemotherapeutic resistance, using FCM to detect the apoptosis of different groups of B-ALL cells. Nrf2 overexpression can protect tumor cells from apoptosis and easily result in apoptosis when Nrf2 expression is repressed. This result is consistent with that of previous studies that used inhibitors. Nrf2 is shown to regulate the sensitivity of tumor cells to VCR therapy by altering their expression rather than the toxicity of inhibitors.

Nrf2 is a transcription factor, and our results establish a new possibility of chemoresistance treatments by targeting this transcriptional network that regulates the expression of apoptotic or anti-apoptotic proteins than the protein itself and using it with traditional chemotherapeutic agents. PI3K-AKT is a classical signal pathway related to apoptosis. In this study, the molecular basis underlying the resistance was investigated. Our data suggest that Nrf2 is regulated by Akt and affects the downstream products of Akt. Proteins in the apoptosis protein family, namely BAD and Bcl-2, are the most affected by Nrf2. Tumor cells with Nrf2 activation may increase antioxidant systems to mitigate oxidative stress, which in turn contributes to anti-apoptotic and drug resistance (54). Nrf2 plays a complex indispensable role in cancer growth and chemoresistance and it has been become a target for the development of anti-cancer drug (55). The influence of Nrf2 beyond redox, such as it can regulated the relation between Ca^2+^ signalling and redox homeostasis, which is correlated with proteins’ sensitive to S-glutathionylation (56). Nrf2, according to regulated ROS levels, other proteins, transcription factors, signal pathways, has been established in cancers. In this study, we characterised the role of Nrf2 in B-ALL apoptotic-dependent chemotherapeutic resistance.

The transcriptional regulatory networks of many genes encoding proteins involved in chemoresistance are well-established; therefore, this approach opens new possibilities for targeted combination therapies (57). To the best of our knowledge, our findings demonstrate, for the first time, a direct relationship between Nrf2 overexpression and anti-apoptotic protein inhibition, BAD, which in the PI3K-AKT pathway that decreased response to chemotherapeutic response in adult patients with B-ALL. Therefore, Nrf2 high expression may be a predictive biomarker of the poor treatment response in adult patients with B-ALL that induction chemotherapy with VCR not effective well. In other words, it may improve the effectiveness of induction chemotherapy, which in turn may allow patients to achieve better remission. In conclusion, our results establish the therapeutic efficacy of a novel combination treatment for adult patients with chemoresistant B-ALL. Hence, novel Nrf2 inhibitors with fewer side effects that could be combined with standard chemotherapeutic agents should be developed for the treatment of drug-resistant haematological malignancies in the future.

## Data Availability Statement

The original contributions presented in the study are included in the article/**Supplementary Material**. Further inquiries can be directed to the corresponding authors.

## Ethics Statement

The animal study was reviewed and approved by Guizhou Laboratory Animal Engineering Technology Center.

## Author Contributions

Funding acquisition, JW. Investigation, LW. Methodology, LW, XL, CF, LC. Project Administration, JW. Resources, JW. Supervision, JW, SW. validation, CP, QK, TZ. Writing—original draft, LW. Writing—review and editing, JW, SW, LW, CP, QK. The author share last authorship, SW. All authors contributed to the article and approved the submitted version.

## Funding

The work is supported by grants from the National Natural Science Foundation of China (NO. 81960032 and NO. 82170168), the Translational Research Grant of NCRCH (2021WWB01 and 2020ZKPB03).

## Conflict of Interest

The authors declare that the research was conducted in the absence of any commercial or financial relationships that could be construed as a potential conflict of interest.

## Publisher’s Note

All claims expressed in this article are solely those of the authors and do not necessarily represent those of their affiliated organizations, or those of the publisher, the editors and the reviewers. Any product that may be evaluated in this article, or claim that may be made by its manufacturer, is not guaranteed or endorsed by the publisher.

## Supplementary Material

Supplementary Figure 1 **(A)** The HO-1 protein levels assessed by western blot. **(B)** The relative gray values were shown in the histogram, *p<0.05.

Supplementary Figure 2 **(A)** The Nrf2 protein levels in Nalm-6, RS4:11, Supb-15 were assessed by western blot. **(B)** The relative gray values were shown in the histogram.
