# Supplementary figures and images for: Nrf2 Overexpression Decreases Vincristine Chemotherapy Sensitivity Through the PI3K-AKT Pathway in Adult B-Cell Acute Lymphoblastic Leukemia
Source: Front Oncol. 2022 May 12;12:876556. doi: 10.3389/fonc.2022.876556 (PMC9134735; doi:10.3389/fonc.2022.876556)

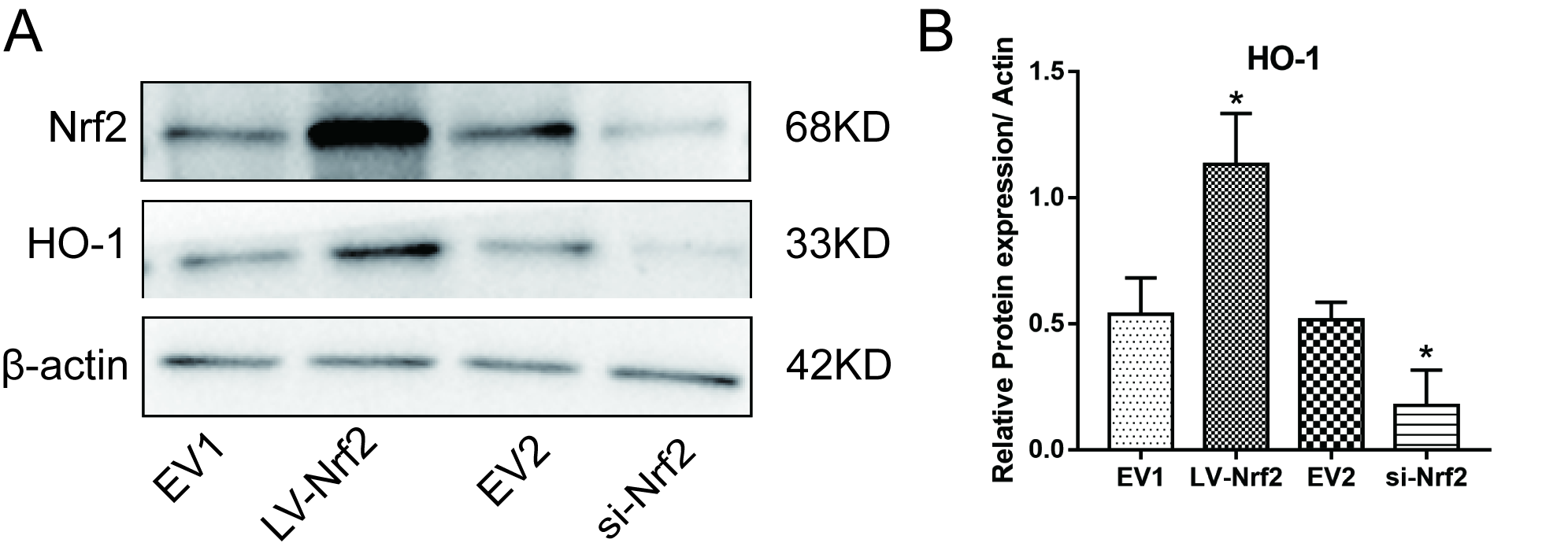

Supplement: Supplementary Figure 1 — (A) The HO-1 protein levels assessed by western blot. (B) The relative gray values were shown in the histogram, *p<0.05. [file Image_1.tif]

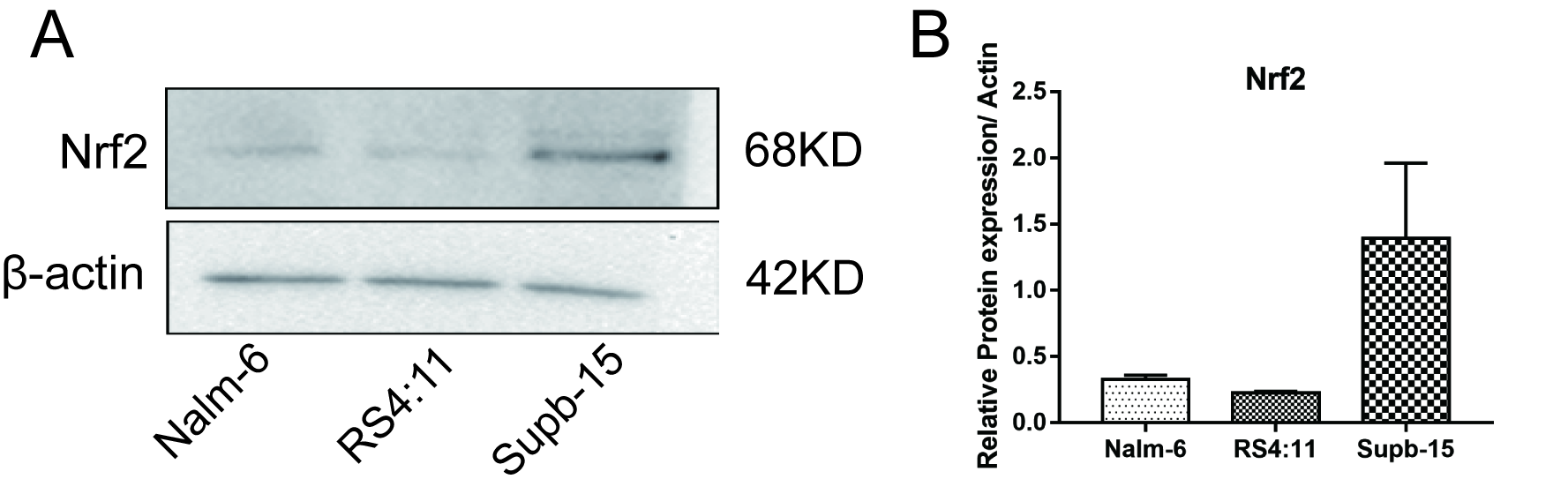

Supplement: Supplementary Figure 2 — (A) The Nrf2 protein levels in Nalm-6, RS4:11, Supb-15 were assessed by western blot. (B) The relative gray values were shown in the histogram. [file Image_2.tif]
